# Does listening to audiobooks affect gait behavior?

**DOI:** 10.1186/s13102-023-00773-6

**Published:** 2023-11-24

**Authors:** Aisuluu Atakanova, Thea Laurentius, Cornelius Bollheimer, Frank Hildebrand, Hannah Lena Siebers

**Affiliations:** 1 Department of Geriatric Medicine, Uniklinik RWTH Aachen, Pauwelsstr. 30, 52074 Aachen, Germany; 2 Department of Orthopedics, Trauma and Reconstructive Surgery, Uniklinik RWTH Aachen, Aachen, Germany

**Keywords:** Gait parameters, Single-task, Dual-task, Treadmill walking, Cognitive performance, Dual-tasking cost

## Abstract

**Background:**

The effect of listening to audiobooks, podcasts, and other audio files while walking on gait performance has not been well studied. Although the number of audio users is growing annually. Evidence suggests that a posture-first strategy contributes to gait stability in healthy individuals during dual-task conditions, but this effect may be diminished when the cognitive task is consciously prioritized. Objectives: To study the effect of listening to an audiobook while walking, as a daily life-like dual-task, on spatiotemporal gait parameters.

**Methods:**

Forty young healthy (24.05 ± 3.66) subjects participated in the study. Spatiotemporal gait parameters were measured for 5 min on a treadmill once without (single-task) and once while listening to an audiobook through over-ear headphones (dual-task). Measured parameters included spatiotemporal parameters, gait phases, maximum pressure, and dual-task cost. Data were statistically analyzed using SPSS software.

**Results:**

There were no significant differences in any of the studied parameters between the single- and dual-task conditions, even though the subjective cognitive load of listening to audiobooks while walking was high. However, participants with different habits had significant differences in gait phases and maximum pressure. Rare listeners had a shorter stance phase, a longer swing phase, and a higher maximum pressure on the dominant heel. They also had significant differences in dual-task costs.

**Conclusion:**

No differences in the spatiotemporal gait parameters for walking with and without listening to audiobooks, as a daily life-like dual-task, were observed. However, the difference between participants who listened rarely and participants who listened often may confirm the “posture first” strategy in young healthy people.

**Trial registration:**

DRKS00025837, retrospectively registered on 23.11.2021.

**Supplementary Information:**

The online version contains supplementary material available at 10.1186/s13102-023-00773-6.

## Introduction

The number of people who listen to audiobooks, voice messages, and podcasts worldwide is growing significantly, with an estimated 278 million listeners in 2019 [1]. The average number of users in Germany in 2019 was 23 million [2]. In many cases, these tasks, such as listening to audiobooks or podcasts, talking on the phone, or texting, were initiated while walking as so-called dual tasks. Performing motor and cognitive tasks at the same time can have a detrimental effect on balance control. This can lead to falls in elderly people [3].

Healthy people usually have no difficulty performing multiple tasks at the same time. They are not aware that this leads to changes in posture. However, behavioral studies have shown that people disproportionately delay their body reactions to external stimuli while performing two simple cognitive tasks simultaneously, as opposed to performing the tasks sequentially [3, 4]. This deceleration in the performance of dual tasks is called the “dual-task interference effect”. According to the literature [5], this dual-task interference effect occurs when information is processed at the central level of decision-making, which only works sequentially. Peripheral levels of decision-making (perceptual and motor) can operate in parallel. In addition, there is evidence that performing a cognitive task may have a negative effect on locomotor function, as the two tasks compete and possibly require the same brain resources [6].

Performing two tasks simultaneously not only leads to competition for brain resources but also forces the brain to prioritize tasks. Prioritizing while walking involves analyzing motor and cognitive tasks in a dual-task condition. Functional reserve and compensatory capacity to perform a double task depend on individual characteristics and previous experience.

The “posture first” strategy refers to healthy individuals who can engage in a concurrent cognitive task while walking. This suggests that healthy subjects may unconsciously prioritize gait stability during dual-task conditions if they have not received specific prioritization instructions [7]. This prioritization can be explained through evolution, as gait stability is more important for individual survival. However, there is evidence that the “posture first” strategy does not always work. Studies have shown that this strategy does not apply for older people [8] and people with various neurological conditions [9].

There have been many studies on the effect of cognitive tasks on gait. However, most of the cognitive tasks (arithmetic or memory tasks) used in previous studies were not reflective of daily practice [10]. After reviewing the literature, it was concluded that the effect of audiobooks, podcasts, and other audio files on gait parameters has been poorly researched, which was the starting point for our study.

The aim of this pilot study was to measure the spatiotemporal gait parameters of young, healthy individuals while engaging in a dual-task condition (simultaneous walking and listening to an audiobook) and to compare these parameters to their performance during a single task (only walking).Therefore, we formulated our alternative hypothesis as follows: listening to audiobooks as a daily life-like dual-task could influence spatiotemporal gait parameters.

## Methods

This cross-sectional study included healthy participants aged between 18 and 35 years. The exclusion criteria were as follows: any chronic disease (e.g., COPD, asthma, hypertension, diabetes) treated with medication; any hearing and/or severe visual impairment; pregnancy; body mass index (BMI) ≥ 35 kg/m², BMI was calculated using weight and height (BMI = weight in kg/(height in meters²)); any acute or chronic pain (e.g., osteoarthritis, after injury); participants with amputation of any limb; and participants using any walking aids (e.g., walker, walking stick). To assess physical and mental health, subjects self-rated their condition on a scale of 1 to 10, with 1 being very poor and 10 being very good.

Measurements were made on a standardized and study-approved treadmill that is an integral part of the Motion Analysis Laboratory in the Department of Geriatric Medicine (type: Zebris FDM-THQM3i, CE certified). Parameters were recorded by the participants at a self-selected speed. A self-selected speed was measured on the ground using a stopwatch and markers. The speed was then transferred to the treadmill. Each participant was warmed up on the treadmill for 1 min before starting the recording. The ball test was used to determine the dominant leg for each subject [11]. Data from this leg were used for statistical processing.

Spatiotemporal parameters such as duration of the stance and swing phase, loading response, mid and terminal stance, pre-swing phase time, step speed, step frequency, step width, step length, and step time were analyzed. Coefficients of variation “CoV” [%] = (standard deviation/mean) *100 were calculated for step time, step length, step width and cadence, indicating the variability between individual steps. In addition, the average of the maximum force achieved in Newtons and the maximum pressure in Newtons per square centimeter were calculated for the forefoot, midfoot, and heel. These parameters were measured with the help of an instrumented treadmill while walking, once for 5 min with over-ear headphones without audio, as the headphones themselves can influence gait parameters, and once for 5 min with an audiobook through the over-ear headphones.

Before the measurement with the audiobook, the subjects were informed about a post-test to check the played information, so that the subjects concentrate on the audiobook and do not concentrate too much on walking. However, the test is not administered at the end.

The order of the two measures, with and without listening to the audiobook, was determined through a simple randomization process. Prior to the experiment, 40 numbers were written down and participants were randomly assigned a number between 1 and 40. Those who drew a number between 1 and 20 were measured with the audiobook first (dual-task condition), while participants who drew a number between 21 and 40 were measured without the audiobook first (single-task condition).This randomization ensured an equal number of subjects for comparison.

Questions about the participants’ behavior while listening to audio and walking during daily life were used to determine how often and for how long the subjects listened to audio files. The participants were divided into frequent and infrequent users by asking, “How often do you listen to audio while walking in your daily life?” There were 4 choices: 1) I do not listen to audio; 2) rarely (1–4 times a month); 3) often (2–3 times a week); and 4) very often (more than 3 times a week). The difference in spatiotemporal gait parameters between participants who listened to audio rarely and those who listened to audio often was calculated. Participants who did not listen to audio and those who listened to audio very often were excluded from the analysis, as they were categorized as extremes. All participants listened to an audiobook of classical literature.

We also used the NASA Task Load Index (NASA-TLK), a standardized questionnaire designed to assess perceived workload [12]. Subjects were asked to complete this questionnaire after each measurement with and without listening to the audiobook. In the analysis, the participants were divided according to the NASA scale into those who experienced “little” subjective load (NASA ≤ 33) and those who experienced “high” subjective load (NASA ≥ 34) while listening to an audiobook while walking.

### Statistical methods

The Zebris FDM-THQM3i treadmill is equipped with proprietary software (Zebris FDM software) for the data derivation. The statistical analysis was prepared using SPSS 26.0 for Windows. The gait parameters are presented as the mean values with standard deviation over the entire measurement period of 5 min each. The results of the Shapiro-Wilk test indicated that the data were normally distributed (Appendix 2). The distribution of data for gait phases and foot pressure was not normal for the whole sample (n = 40), but with a sufficient number (n > 30) of subjects, we made a further calculation than for normally distributed data. Therefore, the paired t-test was used to compare single- and dual-task conditions, with a significance level of p ≤ 0.05. In addition, the dual-tasking costs (DTCs) for individual parameters were calculated. DTC is the cost of performing several tasks at the same time, in our case two tasks - one motor and one cognitive. DTC was calculated according to the following formula: DTC (%) = (100 * (single-task score – dual-task score)/single-task score).

The study was approved by the Ethics Committee of the Faculty of Medicine at RWTH Aachen University (approval number: EK 310/21), and informed consent was obtained from all participants. Data collection for this study started in November 2021 and was finalized in February 2022 and study results are reported according to the CONSORT statement.

## Results

The study included 40 young, healthy subjects without relevant chronic diseases that could affect gait parameters. The general characteristics of the subjects are shown in Table 1.

**Table 1:** General characteristics of the study participants

Characteristics	Frequency
Women	29 (72.5%)
Right leg dominant	38 (95%)
Native speaker	31 (77.5%)
Secondary education (years)	4.13 ± 2.36
Age (years)	24.05 ± 3.66
Weight (kg)	66.01 ± 12.98
Height (cm)	172.30 ± 8.39
Body Mass Index	22.11 ± 3.65
Physical health (scaled from 0 to 10)	8.70 ± 0.96
Physical fitness (scaled from 0 to 10)	7.33 ± 1.13
Mental health (scaled from 0 to 10)	8.43 ± 1.40
Average speed on a treadmill (m/sec)	1.11 ± 0.49

80% of respondents listen to audio in their daily lives, and 32% listen while exercising. A total of 14.5% (n = 7) of the participants answered that they listened to audio while walking very often, 15% (n = 6) answered often, 140% (n = 16) answered rarely and 27.5% (n = 11) answered never.

There were no significant differences in the spatiotemporal gait parameters between the single- and dual-task conditions and no difference between participants who combined listening to audio with walking rarely and often (Table 2). The significant difference coefficients of variation were only for step width between participants, who listened rarely and often.

**Table 2:** Spatiotemporal gait parameters in single vs. dual-task and for rarely vs. often listeners in dual-task

Parameter	Single-task (n = 40)	Dual-task (n = 40)	p	Listen rarely (n = 16)	Listen often (n = 6)	p
Step time, s						
Mean ± SD	0.59 ± 0.50	0.59 ± 0.48	0.452	0.59 ± 0.03	0.59 ± 0.03	0.590
CoV (%) ± SD	4.94 ± 6.30	4.07 ± 5.33	0.253	4.23 ± 6.52	2.88 ± 1.63	0.455
Double step time, s						
Mean ± SD	1.18 ± 0.09	1.18 ± 0.08	0.421	1.17 ± 0.1	1.18 ± 0.06	0.662
CoV (%) ± SD	5.03 ± 7.25	3.29 ± 4.08	0.096	3.23 ± 4.71	2.43 ± 1.99	0.583
Cadence, steps/min						
Mean ± SD	101.99 ± 8.1	102.17 ± 7.6	0.893	103.04 ± 5.9	101.67 ± 5.7	0.631
CoV (%) ± SD	3.01 ± 2.37	2.46 ± 1.82	0.245	2.40 ± 1.94	2.07 ± 1.30	0.674
Step length, cm						
Mean ± SD	63.51 ± 6.2	63.49 ± 6.3	0.992	63.55 ± 5.4	61.03 ± 7.2	0.407
CoV (%) ± SD	2.23 ± 1.35	2.00 ± 1.24	0.441	1.92 ± 1.04	2.49 ± 1.07	0.171
Double step length, s						
Mean ± SD	127.35 ± 12.10	127.09 ± 12.28	0.463	127.3 ± 10.6	121.8 ± 11.9	0.294
CoV (%) ± SD	2.98 ± 2.05	2.64 ± 1.92	0.223	2.95 ± 2.22	2.69 ± 1.86	0.792
Step width, cm						
Mean ± SD	9.01 ± 3.30	9.04 ± 3.59	0.965	9.36 ± 3.6	9.3 ± 1.5	0.971
CoV (%) ± SD	30.23 ± 11.93	29.49 ± 11.48	0.779	32.16 ± 12.74	22.41 ± 6.30	0.029

SD- standard deviation, CoV coefficient of variation

The gait phases show no significant difference between the single- and dual-task conditions. However, there was a significant difference between subjects who were rare listeners and those who were often listeners. Participants who rarely listened to audio while walking had a longer swing phase of 35.59% vs. 34.07% (p < 0.05) and a shorter stance phase, respectively, by shortening the terminal stance and pre-swing (Table 3).

**Table 3:** The gait phases in single and dual tasks and for rare and often listeners in dual tasks

Parameters	Single-task (n = 40)	Dual-task (n = 40)	p	Listen rarely (n = 16)	Listen often (n = 6)	p
Stance phase, %	64.87	64.9	0.926	64.4	65.92	0.032
Loading response, %	14.99	15.03	0.888	14.55	16.07	0.102
Mid stance, %	34.96	34.82	0.793	35.29	33.84	0.079
Terminal stance, %	14.96	15.04	0.817	14.55	16.07	0.037
Pre swing, %	29.93	30.07	0.848	29.09	32.08	0.032
Swing phase, %	35.13	35.10	0.926	35.59	34.07	0.048

As before, no significant difference was measured between the single- and dual-task conditions for maximum pressure. However, the participants who listened to audio rarely in daily life had greater mean maximum forefoot and hindfoot pressures than those who listened often (Table 4).

**Table 4:** Maximum force and pressure of the feet during walking in single vs. dual-task and for rarely vs. often listeners during dual-task

Parameter	Single-task (n = 40)	Dual-task (n = 40)	p	Listen rarely (n = 16)	Listen often (n = 6)	p
Maximum pressure forefoot, N/cm2	20.0 ± 5.0	20.1 ± 5.1	0.982	23.32 ± 5.9	16.5 ± 3.8	0.041
Maximum pressure midfoot, N/cm²	15.8 ± 5.1	15.5 ± 4.6	0.744	15.67 ± 2.9	13.1 ± 2.3	0.053
Maximum pressure hindfoot, N/cm²	17.9 ± 6.2	17.8 ± 6.0	0.960	19.66 ± 5.7	13.4 ± 1.7	< 0.001

N/cm², Newton/cm²

**Table 5:** Dual-tasking costs for spatiotemporal parameters

Parameter	Listen rarely (n = 16)	Listen often (n = 6)	p
DTC step length, %	-0.25 ± 2.15	1.11 ± 1.83	0.092
DTC double step length, %	-0.006 ± 2.13	1.57 ± 1.75	0.054
DTC cadence, %	0.72 ± 1.76	-2.63 ± 2.19	0,022
DTC step time, %	-0.79 ± 1.95	2.36 ± 2.36	0.030
DTC double step time, %	-0.77 ± 1,86	2.72 ± 2.25	0.021

DTC- dual-tasking cost

**Fig. 1:**
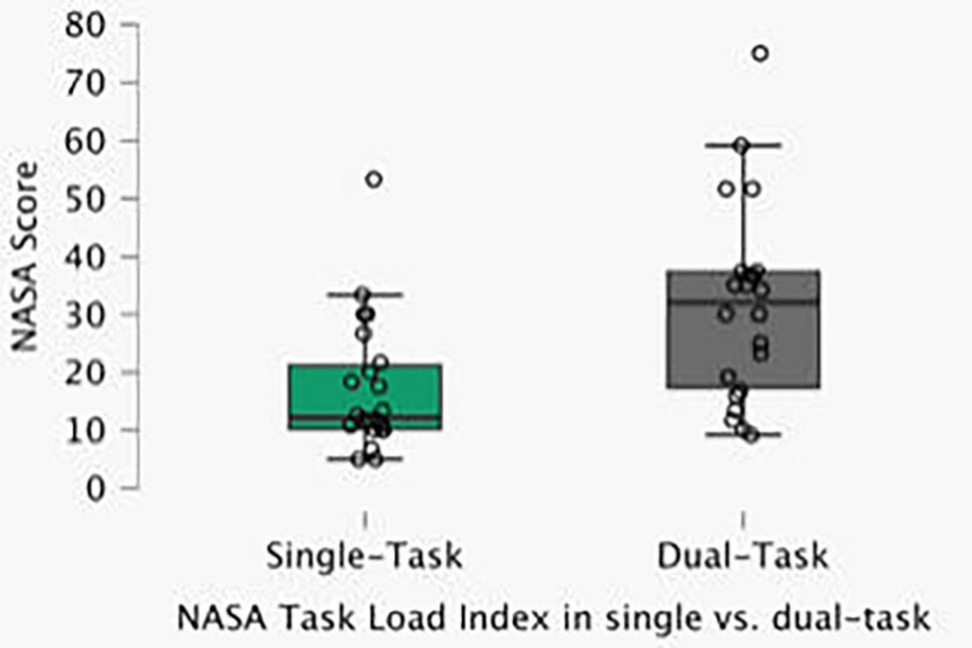
NASA TASK load index in single vs.dual-task

**Fig. 2:**
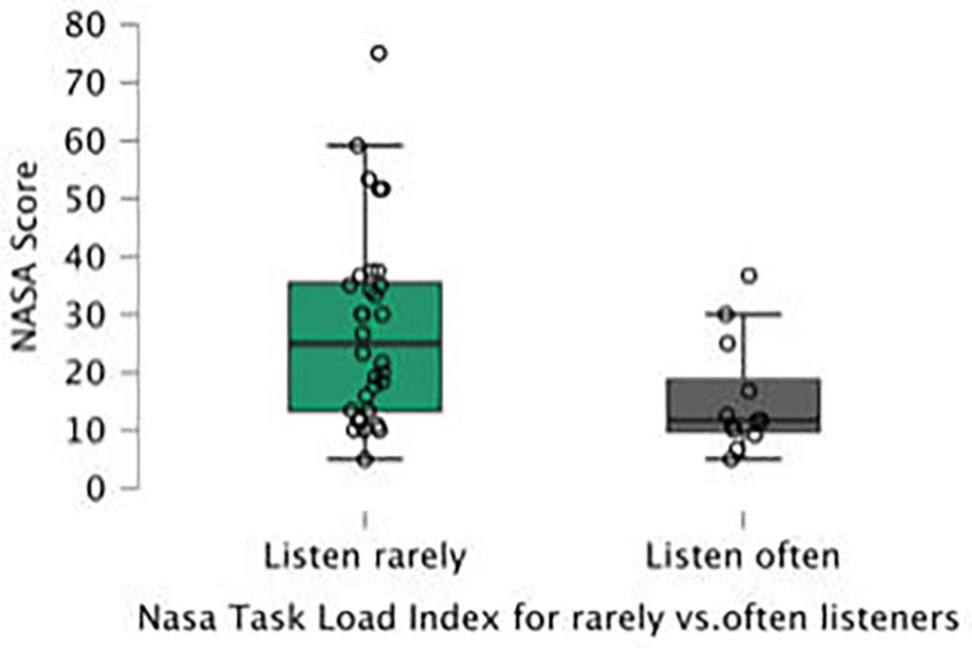
NASA TASK load index for rarely vs.often listeners in dual-task

Listening to an audiobook as a cognitive load, measured by the standardized NASA-TLX questionnaire, was significant for all participants, including those who listened to audiobooks in their daily lives (Figs. 1 and 2).

The mean of the NASA scale was 15.70 ± 9.89 for the single task, which was significantly lower than that for the dual task (28.06 ± 14.64) (p < 0,0001). Participants who listened often had a significantly lower subjective load (21.53 ± 10.83) than participants who listened rarely (35.31 ± 17.56) (p = 0,022).

Participants (n = 25) who experienced “low” subjective load as measured by the NASA TLX did not differ in spatiotemporal gait parameters from those (n = 15) who experienced “high” subjective load while listening to an audiobook and walking (Appendix 1).

Table 5 shows the mean DTC and standard deviations for the spatiotemporal gait parameters for rare and often audio listeners. The DTC was significant for cadence and step time. A positive value for cadence indicates that the value of the measure decreased in the dual-task condition. At a fixed self-paced speed on the treadmill, stride length and stride time were longer and, of course, cadence was lower in the dual task for the infrequent listeners.

The dual-task costs for step length and step time are represented by a negative number for rare listeners, meaning that the step length and step time were longer on the dual task for participants who listened to audio files rarely.

## Discussion

The current study examined and compared the spatiotemporal gait parameters of young healthy subjects during dual-task and single-task conditions. Our results do not support the hypothesis that listening to audiobooks as a daily life-like dual task could affect spatiotemporal gait parameters. However, our results support the hypothesis that listening to audiobooks as a dual task can be learned and that gait parameters may depend on personal habits.

The presented results underline that there are no relevant differences in spatiotemporal gait parameters between single-task and dual-task, such as listening to audiobooks in young adults. This is in line with the “posture first” strategy for young people found in the literature [7]. It is interesting to note that there was no difference in spatiotemporal gait parameters depending on the subjective load measured by the NASA scale. The gait parameters remained unchanged regardless of the level of subjective load of listening to audiobooks in young healthy subjects, indirectly confirming the “posture first” theory.

Significant differences between people with different listening habits were presented. This may be indicative of a learning or habituation process in terms of managing motor tasks and listening simultaneously. Participants who rarely listened to audio files while walking had a shorter stance and a longer swing phase. Although the changes in the gait cycles are not pathological, they are still significantly different, so listening to audiobooks as a dual task may change the gait cycles.

Participants who listened to audio files rarely exerted significantly more pressure on the forefoot and hindfoot. These results may indicate that the forces needed to stabilize the ankle are increased in young people during dual-tasking. These findings are consistent with data from Niederer et al. [13], where reading and researching on a smartphone while walking was also associated with increased hindfoot pressure. These findings are partially consistent with the data from Kondo et al. [14], where there was a tendency for smartphone use to put more pressure on the forefoot than on the hindfoot. Thus, listening to an audiobook as a dual task may affect gait stability by altering the pressure on the foot.

Although no significant difference was found for spatiotemporal gait parameters, the DTC was significant for cadence and step time. It is generally accepted that the DTC of gait parameters is higher in older adults than in younger adults. In addition, many studies have measured higher DTC for gait parameters with smartphone use [14, 16]. At a fixed self-paced speed on the treadmill, stride length and stride time were longer, and cadence was naturally lower in the dual-task for the participants who listened rarely than for participants who listened often to audio files in their daily life. We can therefore say that listening to audiobooks is a daily life-like dual-task, even for young people, and that DTC is significantly different for often and rarely listeners.

## Research implications

Listening to semantically loaded audio files while walking has been poorly studied, although the field has developed significantly in recent years. Our data show the effects of audio files on the gait parameters, such as altering the pressure on the foot and the changes in the gait cycles, only in young subjects with “little experience”. This effect of listening to audio while walking could be a risk factor for falls, especially for elderly people and people with neurological disorders, such as Parkinson’s disease and multiple sclerosis. These effects need to be studied more closely.

### Limitations

These data were generated using an accessible sample group of young people with a narrow age range, which may lead to reduced generalizability of the findings. Further research with a larger number of participants and across a wider range of ages is needed for a robust data. We included only young, healthy adults, and the sample size was small, in part due to restrictions during the coronavirus epidemic in 2021. However, our study used a large number of steps during walking. Treadmill walking differs from real walking due to the inability to voluntarily change gait speed and reduced stride variability, which may make it difficult to extrapolate results to real life. However, we tried to create conditions that were close to daily life, allowing the subjects to choose a comfortable walking speed.

## Conclusions

Even in healthy young people with little previous experience of listening to audio in their daily lives, a simple task such as listening to an audiobook can affect gait phases and hindfoot pressure and requires more dual-task cost. With the increasing amount of audio information available worldwide, further research on dual-task gait changes is needed.

### Electronic supplementary material

Below is the link to the electronic supplementary material.

Supplementary Material 1

Supplementary Material 2

Supplementary Material 3

## Data Availability

The data used and analyzed in the current study are available from the corresponding author upon reasonable request.

## Abbreviations

BMI: Body Mass Index
DTC: Dual-Tasking Costs
EK: Ethics Committee
NASA: TLX-NASA Task Load Index
