# Supplementary material for: Does listening to audiobooks affect gait behavior?
Source: BMC Sports Sci Med Rehabil. 2023 Nov 24;15:159. doi: 10.1186/s13102-023-00773-6 (PMC10675893; doi:10.1186/s13102-023-00773-6)
Supplement: Supplementary file 1 — Supplementary Material 1 [file 13102_2023_773_MOESM1_ESM.pdf]

Appendix 1.

Parameter	NASA Score>33 (n=15)	NASA Score < 33 (n=25)	p
Step time, s	0,60±0,04	0,59±0,04	0,201
Double step time, s	1,12±0,08	1,16±0.09	0,215
Cadence, steps/min	100,17±6,98	103,36±7,8	0,191
Step length, cm	63,21±4,36	63,67±7,33	0,808
Double step length, s	126,55±9,66	127,41±13,79	0,819
Step width, cm	9,78±5,06	8,59±2,33	0,405
