# Supplementary material for: Does listening to audiobooks affect gait behavior?
Source: BMC Sports Sci Med Rehabil. 2023 Nov 24;15:159. doi: 10.1186/s13102-023-00773-6 (PMC10675893; doi:10.1186/s13102-023-00773-6)
Supplement: Supplementary file 2 — Supplementary Material 2 [file 13102_2023_773_MOESM2_ESM.pdf]

Shapiro-Wilk test for normality of data

(for the whole sample, n=40)

**Parameter**	**Mean**	**Standard deviation**	**Minimum**	**Maximum**	**p**
Step time, s	0,59	0,045	0,51	0,69	0,619
Double step time, s	1,18	0,089	1,02	1,37	0,406
Cadence, steps/min	102,1	7,65	87,47	117,61	0,729
Step length, cm	63,54	6,22	50,64	75,25	0,445
Double step length, s	127,15	12,16	102,12	150,56	0,708
Step width, cm	2,4	0,64	1,28	3,92	0,757
**Gait cycle**					
Stance phase, %	64,95	1,66	62,58	70,1	<0,001
Loading response, %	15,02	1,67	12,81	20,69	<0,001
Mid stance, %	34,82	1,77	29,95	37,07	0,003
Terminal stance, %	15,1	1,77	12,54	19,78	0,005
Pre swing, %	30,11	3,35	25,5	39,7	0,01
Swing phase, %	35,05	1,66	29,9	37,42	<0,001
**Footpressure**					
Maximum pressure forefoot, N/cm2	20,07	5,08	12,02	32,88	0,035
Maximum pressure midfoot, N/cm^2	15,5	4,61	10,36	35,51	<0,01
Maximum pressure hindfoot, N/cm^2	17,83	6,01	10,22	35,87	<0,01

Shapiro-Wilk test for normality of data

(Group “Listen rarely“, n=16)

Parameter	Mean	Standard deviation	Minimum	Maximum	p
Step time, s	0,58	0,03	0,53	0,66	0,959
Double step time, s	1,16	0,07	1,07	1,31	0,568
Cadence, steps/min	103,04	5,98	91,8	112,3	0,766
Step length, cm	63,55	5,4	52,5	72,68	0,511
Double step length, s	127,3	10,58	106,47	143,59	0,792
Step width, cm	9,35	3,57	3,86	17,3	0,682
**Gait cycle**					
Stance phase, %	64,4	1,32	62,58	67,24	0,431
Loading response, %	14,55	1,27	12,81	17,37	0,182
Mid stance, %	35,29	1,42	31,93	37,07	0,131
Terminal stance, %	14,55	1,43	12,54	17,94	0,467
Pre swing, %	29,09	2,67	25,5	35,31	0,266
Swing phase, %	35,59	1,32	32,77	37,42	0,431
**Footpressure**					
Maximum pressure forefoot, N/cm2	21,32	5,93	14,32	32,88	0,069
Maximum pressure midfoot, N/cm²	15,67	2,93	10,36	20,48	0,324
Maximum pressure hindfoot, N/cm²	19,66	5,77	11,39	30,6	0,175
**Dual-tasking costs for spatiotemporal parameters**					
DTC step length, %	-0,25	2,15	-4,19	4,53	0,12
DTC double step length, %	-0,0006	2,13	-3,24	4,65	0,099
DTC cadence, %	0,72	1,76	-1,87	4,29	0,065
DTC step time, %	-0,25	2,15	-4,19	4,53	0,118
DTC double step time, %	-0,77	1,85	-4,67	1,73	0,058

Shapiro-Wilk test for normality of data

(Group “Listen often“, n=6)

Parameter	Mean	Standard deviation	Minimum	Maximum	p
Step time, s	0,59	0,03	0,55	0,62	0,082
Double step time, s	1,19	0,07	1,1	1,27	0,337
Cadence, steps/min	101,26	6,2	94,25	109,31	0,242
Step length, cm	61,31	6,61	50,64	68,39	0,598
Double step length, s	122,23	11,12	106,32	132,66	0,25
Step width, cm	9,44	1,67	7,94	11,65	0,106
**Gait cycle**					
Stance phase, %	66,25	2,18	63,49	70,1	0,378
Loading response, %	15,94	2,65	13,11	20,69	0,368
Mid stance, %	33,88	2,09	30,4	36,67	0,848
Terminal stance, %	16,42	1,79	13,71	19,01	0,931
Pre swing, %	32,37	4,25	26,82	39,7	0,567
Swing phase, %	33,74	2,18	29,9	36,51	0,378
**Footpressure**					
Maximum pressure forefoot, N/cm2	16,61	3,71	12,02	22,89	0,788
Maximum pressure midfoot, N/cm^2	13,25	2,18	10,92	15,82	0,228
Maximum pressure hindfoot, N/cm^2	13,39	1,68	10,65	15,58	0,909
**Dual-tasking costs for spatiotemporal parameters**					
DTC step length, %	1,49	1,88	-0,58	3,63	0,175
DTC double step length, %	1,72	1,6	-0,13	3,52	0,293
DTC cadence, %	-2,61	1,95	-5,7	0,08	0,951
DTC step time, %	1,49	1,88	-0,58	3,63	0,174
DTC double step time, %	2,68	2,01	-0,08	5,7	0,852
